# Adeno‐Associated Virus Vector‐Mediated Expression of Neuroligin 2 in the Mouse Hippocampus Attenuates the Progression of Pentylenetetrazol‐Induced Seizures

**DOI:** 10.1002/jgm.70116

**Published:** 2026-10-07

**Authors:** Tomohiro Tokutake, Akane Hara, Ayano Murayama, Yuto Mitsuda, Jun Yokose, Yusuke Yano, Yuki Shigetsura, Mika Ito, Naomi Takino, Shinichi Kumagai, Kuniko Shimazaki, Shin‐Ichi Muramatsu, Atsumi Nitta

**Affiliations:** ^1^ Department of Pharmaceutical Therapy & Neuropharmacology, Faculty of Pharmaceutical Sciences University of Toyama Toyama Japan; ^2^ Laboratory of Mathematical Medicine and Pharmaceutical Assessment, Faculty of Pharmaceutical Sciences University of Toyama Toyama Japan; ^3^ Department of Clinical Pharmacology and Therapeutics Kyoto University Hospital Kyoto Japan; ^4^ Division of Neurological Gene Therapy, Center for Open Innovation Jichi Medical University Shimotsuke Japan; ^5^ Department of Neurosurgery Jichi Medical University Shimotsuke Japan

**Keywords:** adeno‐associated virus, epilepsy, hippocampus, neuroligin 2, pentylenetetrazol, seizure

## Abstract

Gene therapy can target specific brain regions and may be an effective therapeutic tool for drug‐resistant epilepsy. Neuroligin 2 (NL2) is a postsynaptic cell‐adhesion protein involved in the formation of inhibitory synapses. Since epileptic seizures are attributed to excessive brain neuronal activity, we hypothesized that NL2‐mediated inhibitory signaling suppresses seizures. Male C57BL/6J mice were bilaterally injected into the hippocampus (HPC) with an adeno‐associated virus (AAV) vector expressing NL2 under the control of synapsin I promoter (AAV‐NL2). Mice treated with AAV‐NL2 were intraperitoneally administered with 35 mg/kg pentylenetetrazol (PTZ) every other day. Ordinal logistic regression analysis revealed that NL2 expression exerted a protective effect against seizure progression induced by repeated PTZ administration. In addition, ventral HPC c‐Fos protein levels were significantly suppressed with AAV‐NL2 injection. NL2 expression induced by intra‐HPC AAV‐NL2 injection attenuates the progression of PTZ‐induced seizures and may represent a potential target for seizure control.

## Graphical abstract

Mice received bilateral hippocampal injections of an AAV vector expressing NL2, followed by repeated PTZ administration. NL2 expression in the HPC attenuated seizure progression and reduced c‐Fos expression in the ventral HPC. These findings suggest that AAV‐mediated NL2 expression in the HPC may be a potential therapeutic strategy for seizure control.

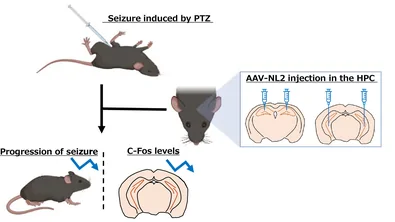

**Abbreviations**
AAV: adeno‐associated virus
CI: confidence interval
GABA: gamma‐aminobutyric acid
GAPDH: glyceraldehyde‐3‐phosphate dehydrogenase
GFP: green fluorescent protein
HPC: hippocampus
HRP: horseradish peroxidase
i.p.: intraperitoneal
NL2: neuroligin 2
OR: odds ratio
PBS: phosphate‐buffered saline
PFA: paraformaldehyde
PTZ: pentylenetetrazol
vg: vector genome

## 1 Introduction

Epilepsy, characterized by spontaneous and recurrent seizures, is one of the most common neurological disorders [1]. Approximately 30% of patients are resistant to existing anti‐seizure medications [2, 3], most of whom suffer from poorly controlled seizures in the long term [4]. Surgical resection of the epileptic focus may be an effective treatment option for drug‐resistant cases [5]. However, resective surgery is not always feasible, especially in cases of multifocal epilepsy or when the focus involves eloquent areas.

Gene therapy can specifically target the epileptic focus while preserving its structural integrity to overcome these limitations [6, 7]. Adeno‐associated virus (AAV) vectors are valuable tools for gene transfer into nondividing cells, including neurons, enabling long‐term gene expression [8, 9]. The clinical safety and efficacy of intraparenchymal AAV delivery have already been demonstrated in other neurological disorders, such as Parkinson's disease and aromatic amino acid decarboxylase deficiency [10, 11, 12, 13, 14, 15].

Neuroligin 2 (NL2) is a cell‐adhesion molecule localized at inhibitory synapses [16] that plays a key role in their formation [17, 18]. The neuroligin family binds to presynaptic neurexins [19, 20]. NL2 anchors the gamma‐aminobutyric acid type A (GABA_A_) receptor to a postsynaptic scaffold using gephyrin and collybistin [21]. NL2‐knockout mice exhibit decreased inhibitory signaling in the hippocampus (HPC) [22, 23]. Epileptic seizures are induced by neuronal hyperexcitability [24]. Enhancing inhibitory signaling through NL2 expression in epileptic foci may suppress pathological excitation. We previously reported that the systemic delivery of an AAV vector expressing NL2 under the control of the synapsin I promoter (AAV‐NL2) through intracardiac injection inhibited seizures in a mice model of epilepsy [25]. However, the anti‐seizure effects of local AAV‐NL2 injections remain unclear, and validating their efficacy is crucial for the future clinical translation of this therapeutic approach.

We utilized the pentylenetetrazol (PTZ)‐induced seizure model, which is widely used to evaluate seizure susceptibility, to evaluate the efficacy of focal NL2 gene delivery [26, 27]. PTZ enhances neuronal excitability and triggers seizures by antagonizing GABA_A_ receptors [28]. As the HPC is one of the most sensitive regions to PTZ [29, 30], we hypothesized that intra‐HPC NL2 expression would inhibit PTZ‐induced hyperexcitability and seizures. Male C57BL/6J mice received an intra‐HPC injection of AAV‐NL2, followed by the intraperitoneal (i.p.) administration of PTZ, and the protective effects of NL2 against seizures were assessed using behavioral and biochemical approaches.

## 2 Methods

### 2.1 Animals

Six‐week‐old male adult C57BL/6J mice were obtained from Nihon SLC (Hamamatsu, Japan). Mice were maintained under controlled conditions (23°C ± 2°C), humidity (50% ± 5%), and a 12‐/12‐h light/dark cycle (light cycle, 7:00–19:00). Mice were given water ad libitum and standard rodent chow (CE‐2; CLEA Japan Inc., Tokyo, Japan). Mice were acclimatized to the environment for 1 week before AAV vector injection. Injected mice were transferred to a box with an adjusted light/dark cycle (light cycle, 11:00–23:00) 1 week before evaluation of seizure to enable daytime PTZ administration. Animal experiments were approved by the Animal Care and Use Committee of the University of Toyama (Approval No: A2025PHA14) and were conducted in compliance with the Institutional Animal Experiment Handling Rules of the University of Toyama. The number of animals used was kept to the minimum necessary to obtain reliable and interpretable results.

### 2.2 PTZ Administration for Seizure Induction

The preparation and administration of PTZ (Sigma‐Aldrich, St. Louis, MO, USA) were performed described previously [31, 32]. PTZ was dissolved in 0.9% saline (Otsuka Pharmaceutical, Tokyo, Japan) to a concentration of 1.75 mg/mL on the day of use and was administered (35 mg/kg, i.p.) to mice every other day. The maximum of PTZ administration was 10 times or four times in Figures 2 and 3, respectively.

### 2.3 AAV Vector Production and Microinjection Into the HPC

AAV vectors were constructed and microinjected as described previously [25, 33]. AAV2 vectors expressing mouse NL2 (AAV‐NL2) and green fluorescent protein (GFP) (AAV‐mock) under the control of the synapsin I promoter were generated. Titers of AAV‐NL2 and AAV‐mock were 1.2 × 10^13^ and 1.7 × 10^13^ vector genome (vg) per mL, respectively.

Mice were anesthetized with a mixture of three agents administered i.p. (0.75‐mg/kg medetomidine [domitol], Orion, Espoo, Finland; 4‐mg/kg midazolam, Sandoz, Tokyo, Japan; and 5‐mg/kg butorphanol, Meiji Seika Pharma, Tokyo, Japan) and fixed in a brain stereotaxic apparatus (SR‐5 M, Narishige, Tokyo, Japan). AAV vectors (0.5 μL per site) were bilaterally injected into the dorsal (coordinates from bregma: AP, −1.7 mm; ML, ±1.0 mm; DV, 1.8 mm) and ventral HPC (AP, −3.3 mm; ML, ±3.3 mm; DV, 3.5 mm) at a rate of 0.025 μL/min [34]. PTZ was administered 3 weeks after AAV vector injection to allow for sufficient transgene expression.

### 2.4 Seizure Evaluation

PTZ‐induced seizures were evaluated as described previously [31, 32]. Mice were habituated to the experimental room and observation boxes for 1 h. Immediately after PTZ administration, mice were placed in observation boxes and recorded using a video camera for 30 min. Seizure intensity was scored as follows: 0: normal behavior, no abnormality; 1: immobilization, lying on belly; 2: head nodding, facial, forelimb, or hindlimb myoclonus; 3: continuous whole‐body myoclonus, myoclonic jerks, tail held up stiffly; 4: rearing, tonic seizure, falling on its side; 5: tonic–clonic seizure, falling on its back, wild rushing, and jumping; 6: death. Seizure latency was defined as the time taken to reach a score of ≧ 4 to evaluate severe seizures. Latency was recorded as 30 min for mice that did not reach a score of 4 within the 30‐min observation period.

### 2.5 Fluorescence Imaging

Fluorescence observations were performed as described previously [33]. Mice were administered with a mixture of three agents and then perfused with ice‐cold phosphate‐buffered saline (PBS), followed by fixation in 4% paraformaldehyde (PFA). Brain tissues were postfixed in 4% PFA overnight before cryoprotection in sucrose solutions (10%, 20%, and 30% in PBS). The tissues were embedded in Tissue‐Tek O.C.T. Compounds (Sakura Finetek Japan, Tokyo, Japan) and sectioned at 20 μm thickness using a CM3050S cryostat (Leica, Wetzlar, Germany). GFP expression was detected by its autofluorescence. Fluorescence images were obtained using a BZ‐X800 microscope (Keyence, Osaka, Japan) and ZEISS LSM 780 microscope (Carl Zeiss, Jena, Germany).

### 2.6 Immunohistochemistry

Immunohistochemistry was performed as described previously [33]. The sections were prepared as described for fluorescence imaging. After each incubation step, the sections were washed with PBS containing 0.1% Tween 20 (PBS‐T). The sections were incubated in methanol for 10 min and then in 0.3% Triton X‐100 in PBS for 15 min. For antigen retrieval, the sections were incubated in 10‐mM Tris‐EDTA buffer (pH 9.0) at 95°C for 10 min. The sections were blocked with 10% goat serum (Merck, Darmstadt, Germany) in PBS (blocking buffer, BB) at room temperature for 1 h. The sections were incubated with anti‐gephyrin (1:100, 147 021, Synaptic Systems, Goettingen, Germany) and anti‐NL2 antibody (1:200, 129 203, Synaptic Systems, Goettingen, Germany) in BB at 4°C overnight. The sections were incubated with CF 488 goat anti‐rabbit IgG (H + L) (1:1000; Biotium, Fremont, CA, USA) and CF 594 goat anti‐mouse IgG (H + L) (1:500; Biotium, Fremont, CA, USA) in BB at room temperature for 2 h and with DAPI at room temperature for 10 min. After the final incubation step, the sections were mounted with Fluoromount (Diagnostic BioSystems, Pleasanton, CA, USA). Fluorescence images were obtained using a ZEISS LSM 780 microscope (Carl Zeiss, Jena, Germany).

### 2.7 Quantitative Reverse Transcription (RT‐q) PCR

RT‐qPCR was performed as described previously [35]. HPC tissues treated with AAV vectors were homogenized using ISOGEN (Nippon Gene, Tokyo, Japan). The extracted RNA was processed using DNase (Promega Corporation, Madison, WI, USA) to remove DNA. Reverse transcription was performed on the RNA samples using the PrimeScript RT Reagent Kit (Takara Bio, Kusatsu, Japan) to construct cDNA, which was used as a template for qPCR. The temperature was controlled using a Dice Gradient PCR Thermal Cycler (Takara Bio). The reaction was carried out using THUNDERBIRD SYBR qPCR Mix (Toyobo, Osaka, Japan) on a CFX Connect Real‐Time PCR Detection System (Bio‐Rad, Hercules, CA, USA). Primers mouse NL2 pair (forward: 5′‐TTGCCCACTTTGGAGGTGAC‐3′, reverse: 5′‐ACTTGAGCGGCTGGTAGTTG‐3′) and mouse 36B4 pair (forward: 5′‐ACCCTGAAGTGCTCGACATC‐3′, reverse: 5′‐AGGAAGGCCTTGACCTTTTC‐3′) were used. 36B4 was used as the housekeeping gene.

### 2.8 Western Blotting

Western blotting was performed as described previously [36]. HPC tissues were collected 2 h after PTZ administration and immediately frozen in liquid nitrogen. Tissues were homogenized in lysis buffer. After centrifugation, the supernatant was collected, and the protein concentration was measured using BCA kit (Wako, Tokyo, Japan). Protein solutions were mixed with 2 × loading buffer and denatured at 95°C for 5 min. Protein samples (10 μg per well) were separated by SDS‐polyacrylamide gel electrophoresis using 10% acrylamide gel at 0.02 A and transferred onto a membrane (Millipore, Darmstadt, Germany) at 100 V for 1 h. The membrane was blocked with 5% bovine serum albumin (BSA; Nacalai Tesque, Kyoto, Japan) in Tris‐buffered saline containing 0.1% Tween 20 (TBS‐T) at room temperature for 1 h. The membrane was then incubated with primary antibodies against NL2 (1:1000), beta‐tubulin (1:1000, 2146, Cell Signaling, Danvers, MA, USA), c‐Fos (1:1000, 226 008, Synaptic Systems, Goettingen, Germany) and glyceraldehyde‐3‐phosphate dehydrogenase (GAPDH) (1:1000, M171‐3, MBL, Nagoya, Japan) diluted in 5% BSA in TBS‐T at 4°C overnight. GAPDH and beta‐tubulin were used as housekeeping proteins. After washing, the membrane was incubated with horseradish peroxidase (HRP)‐conjugated secondary antibodies, anti‐rabbit IgG HRP (1:5000, NA9340V, GE Healthcare, Chalfont, UK), and anti‐mouse IgG HRP (1:5000, NA9310V, GE Healthcare, Chalfont, UK), diluted in 5% BSA in TBS‐T at room temperature for 2 h. The signals were detected by SuperSignal West Pico PLUS Chemiluminescent Substrate (Thermo Fisher Scientific, Waltham, MA, USA) and detected with the ChemiDoc Touch MP Imaging System (Bio‐Rad). Density was quantified using ImageJ (National Institutes of Health, Bethesda, MD, USA). The images of all membranes are shown in Figures S1b and S2.

### 2.9 Statistical Analysis

Statistical analyses were conducted using Prism version 9 (Graph Pad Software, San Diego, CA, USA) and SPSS Statistics (IBM, Armonk, NY, USA). All data are expressed as the mean ± standard error of the mean. The unpaired *t‐*test (parametric) or Mann–Whitney *U* test (nonparametric) was used for comparison between the mock and NL2 groups. Statistical significance was set at *p* < 0.05. Gene expression levels in the RT‐qPCR analysis were normalized to 36B4 expression, and protein expression levels in western blot analysis were normalized to GAPDH or beta‐tubulin. Ordinal logistic regression was performed to assess the association between seizure scores and the effects of the AAV‐NL2 injection. The regression coefficients (B) obtained from ordinal logistic regression analysis were exponentiated to calculate EXP (B) values, which were reported as odds ratio (OR). Survival probability between the mock and NL2 group was analyzed by Log‐rank test. Seizure latency was analyzed using two‐way ANOVA followed by Bonferroni's post hoc test. Body weight changes were evaluated using two‐way repeated measures ANOVA, followed by Bonferroni's post hoc test. For these ANOVAs, *F* values were described by the main effects of NL2 (mock vs. NL2) and the main effect of the number of PTZ administrations, as well as their interaction. Correlations between the total seizure scores and protein levels were analyzed using Spearman's rank correlation coefficients. Mice that died during the PTZ administration period were excluded from analysis. In Figure 2, a total of 15 mice were initially included in each of the AAV‐mock and AAV‐NL2 groups. After 10 administrations of PTZ, the numbers of surviving mice were *n* = 1 in the AAV‐mock and *n* = 4 in the AAV‐NL2 groups. In Figure 3, a total of eight mice were initially included in each of the AAV‐mock and AAV‐NL2 groups. After four administrations of PTZ, the numbers of surviving mice were *n* = 8 in the AAV‐mock and *n* = 8 in the AAV‐NL2 groups.

## 3 Results

### 3.1 AAV Vector‐Mediated NL2 Expression in the HPC

We developed AAV vectors expressing NL2 or GFP in neurons (AAV‐NL2 or AAV‐mock expressing GFP) [25], which were bilaterally injected into the dorsal and ventral HPC of mice. Successful injection was confirmed by intrinsic GFP fluorescence in AAV‐mock‐injected mice (Figure 1a–c). RT‐qPCR analysis revealed that NL2 mRNA levels in HPC were significantly higher in the AAV‐NL2 group than in the AAV‐mock group (*t*_6_ = 19.75, *p* < 0.0001; unpaired *t*‐test) (Figure 1d). NL2 protein levels in HPC were significantly higher in the AAV‐NL2 group than in the AAV‐mock group (*t*_6_ = 3.633, *p* < 0.05; unpaired *t*‐test) (Figure 1e). Colocalization of NL2 and gephyrin was confirmed in the HPC of AAV‐NL2‐treated mice (Figure S1a).

**FIGURE 1:**
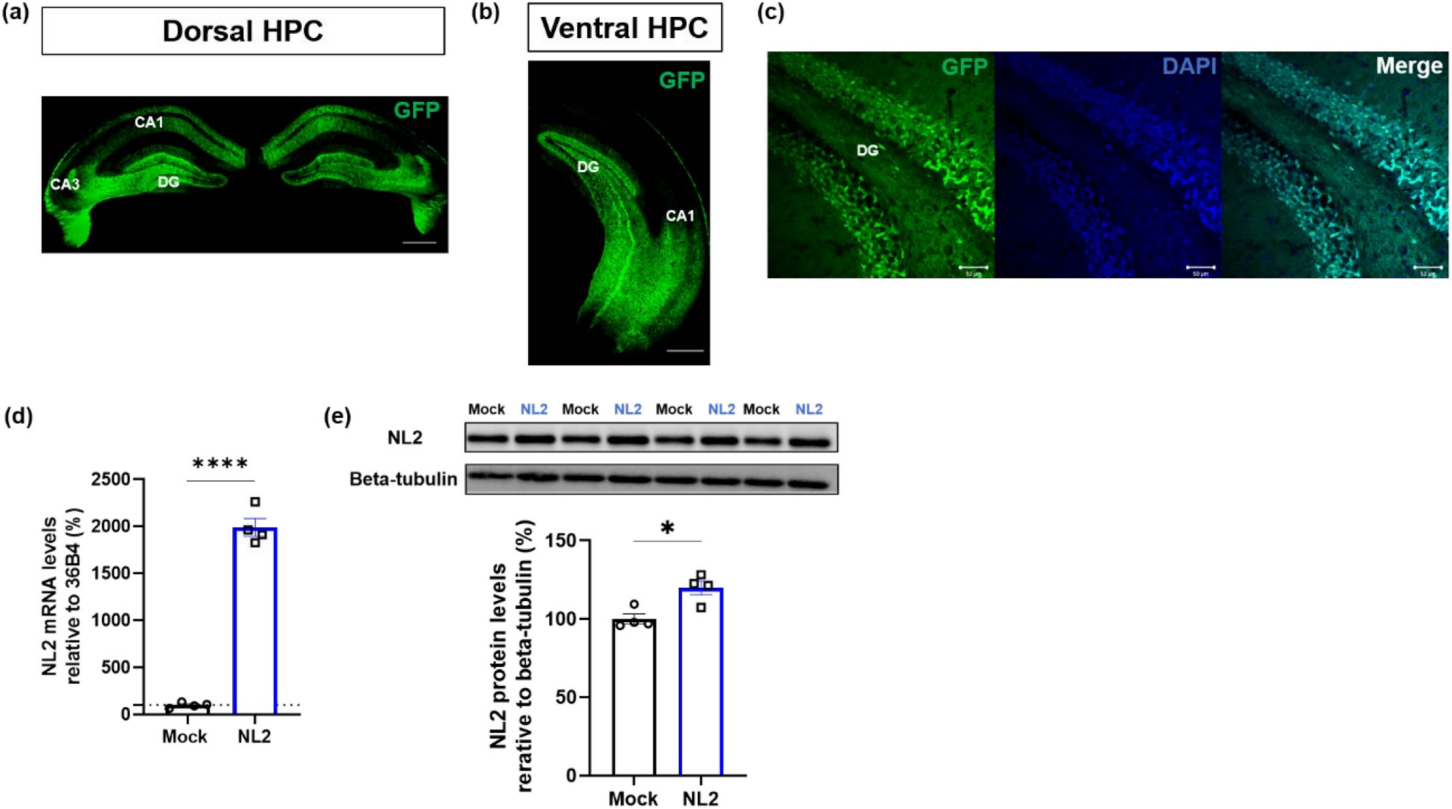
AAV vectors injection site and NL2 expression in the HPC. (a, b) Representative images showing GFP expression at the injection sites in the (a) dorsal and (b) ventral HPC. Scale bars: 500 μm. (c) Representative high‐power field images showing GFP, DAPI, and merge at the injection sites. Scale bars: 50 μm. (d) NL2 mRNA levels relative to 36B4 in the AAV‐mock and AAV‐NL2 groups (*n* = 4 per group; ***p < 0.0001, unpaired *t*‐test). The mean of mock group mRNA levels was represented as 100%. (e) Representative western blots quantification of NL2 and beta‐tubulin in the AAV‐mock and AAV‐NL2 groups (*n* = 4 per group; *p < 0.05, unpaired *t*‐test). The mean of mock group protein levels was represented as 100%. Data are presented as the mean ± SEM. AAV, adeno‐associated virus; HPC, hippocampus; NL2, neuroligin 2.

### 3.2 NL2 Expression in the HPC Inhibited PTZ‐Induced Seizures

Following AAV injection into the HPC, mice were administered PTZ (35 mg/kg, i.p.) every other day (Figure 2a). PTZ‐induced seizures were scored in the AAV‐mock (Figure 2b) and AAV‐NL2 groups (Figure 2c). Ordinal logistic regression was used to evaluate the relationship between seizure scores and HPC NL2 expression. Mice treated with AAV‐NL2 were more likely to display low scores than those treated with AAV‐mock (OR = 2.15, 95% confidence interval = 1.32–3.50) (Figure 2d,e and Table 1). This indicated that seizure progression in the AAV‐NL2 group following repeated PTZ administration was milder than that in the AAV‐mock group. Survival probability was not different between the AAV‐mock and AAV‐NL2 (Figure S3a). Seizure latency to reach a score ≧ 4 was significantly prolonged by NL2 expression at the sixth administration (Figure S3b). Body weights measured during the experiments did not differ between the two groups (Figure S3c). These results suggested that NL2 expression in the HPC suppresses seizure progression induced by repeated PTZ administration.

**FIGURE 2:**
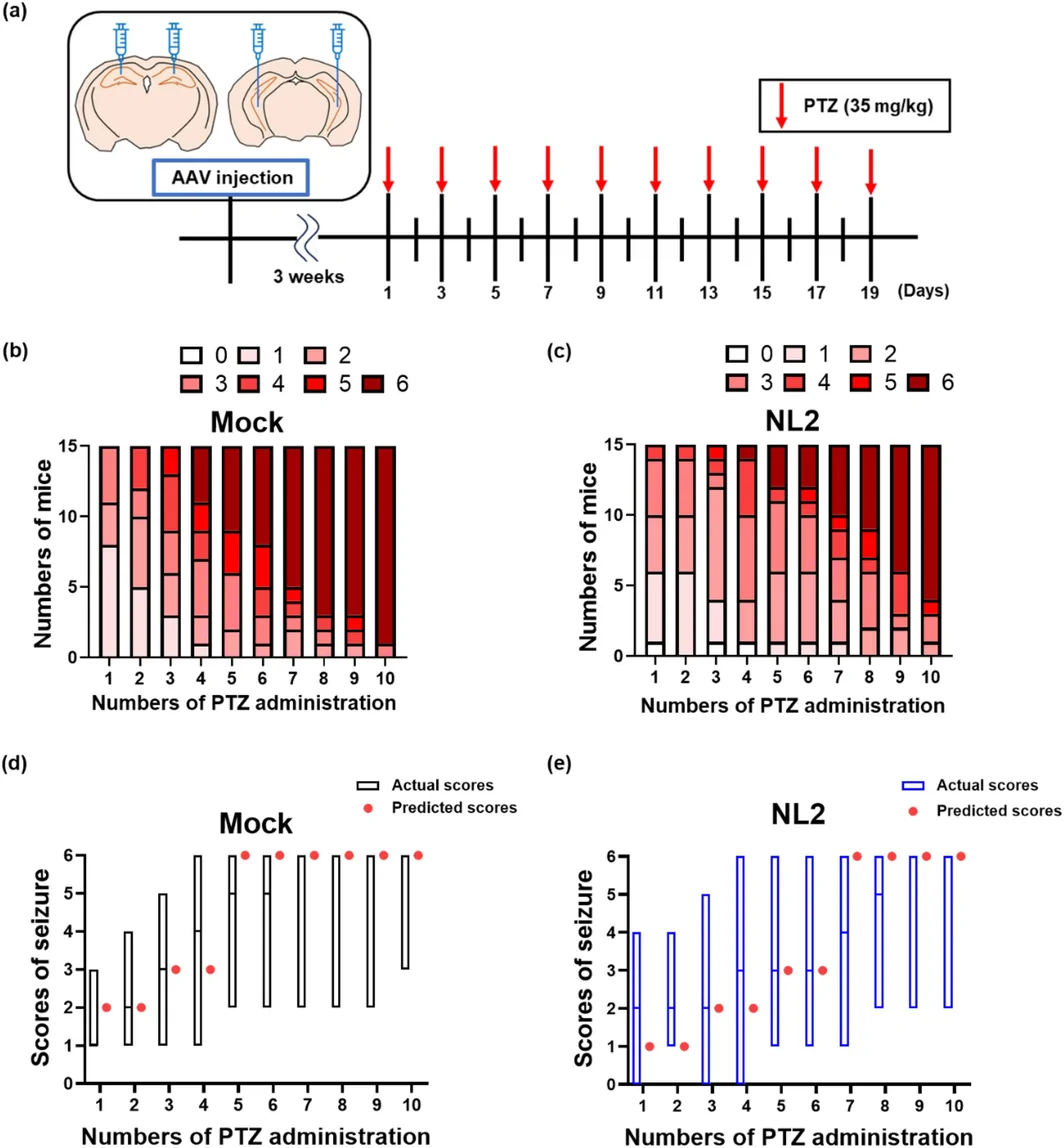
Intra‐HPC expression of NL2 attenuates PTZ‐induced seizure progression. (a) Experimental design. Three weeks after AAV vector injection, the mice were intraperitoneally administered with PTZ (35 mg/kg) 10 times every other day. (b, c) Distribution of seizure stages over PTZ administrations in the (b) AAV‐mock and (c) AAV‐NL2 groups. Death (score: 6) was included to visualize seizure progression (*n* = 15 per group). (d, e) Progression of actual and predicted seizure scores calculated from ordinal logistic regression across PTZ administrations in the (d) AAV‐mock and (e) AAV‐NL2 groups. Predicted values were derived using an ordinal logistic regression model. Box plots represent the maximum, minimum, and mean values. HPC, hippocampus; NL2, neuroligin 2; PTZ, pentylenetetrazol.

**TABLE 1:** The relationship between seizure scores and AAV‐NL2 was analyzed by ordinal logistic regression with SPSS.

	Odds ratio	Wald	*p*	95% confidence interval	
				Lower limit	Upper limit
Scores = 0	0.07	18.59	< 0.001	0.02	0.23
Scores = 1	1.19	0.34	0.559	0.67	2.11
Scores = 2	5.38	30.53	< 0.001	2.96	9.77
Scores = 3	18.41	71.60	< 0.001	9.37	36.16
Scores = 4	41.68	97.82	< 0.001	19.91	87.27
Scores = 5	87.10	117.27	< 0.001	38.82	195.59
PTZ	1.46	53.07	< 0.001	1.32	1.62
NL2 vs. mock	2.15	9.55	0.002	1.32	3.50

*Note:* The six rows labeled with score values correspond to the results of threshold estimation for the classification of seven seizure scores. PTZ and NL2 vs. mock rows correspond to the evaluation of the respective covariates. The estimated coefficients and their 95% CIs were transformed into odds ratio by exponential transformation (**p < 0.01, ordinal logistic regression).

Abbreviations: AAV, adeno‐associated virus; CI, confidence interval; NL2, neuroligin 2; OR, odds ratio.

### 3.3 NL2 Expression Attenuated PTZ‐Induced HPC Activation

Acute and chronic PTZ administration has been reported to increase c‐Fos expression in the HPC, a marker of neuronal activity [37, 38, 39, 40]. We quantified the c‐Fos protein in HPC tissues to assess the protective effects of NL2 against the PTZ‐induced increases in neuronal excitability. Because mice repeatedly treated with PTZ tended to develop severe seizures by the fourth administration, they were sacrificed after this point (Figure 3a). PTZ (35 mg/kg) induced seizures in both the AAV‐mock (Figure 3b) and AAV‐NL2 groups (Figure 3c). NL2 expression in the HPC inhibited total seizures scores (*p* = 0.0295; Mann–Whitney *U* test) (Figure 3d). Compared to the AAV‐mock group, c‐Fos expression in AAV‐NL2‐treated mice tended to decrease in the dorsal HPC (*t*_14_ = 1.981, *p* = 0.0676; unpaired *t*‐test) (Figure 3e) and was significantly reduced in the ventral HPC (*t*_14_ = 3.565, *p* = 0.0031; unpaired *t*‐test) (Figure 3f). A correlation was not observed between seizure scores and levels of c‐Fos expression in both the dorsal and ventral HPC (Figure S4). These results suggested that NL2 expression partially alleviates excessive HPC neuronal activity induced by PTZ administration.

**FIGURE 3:**
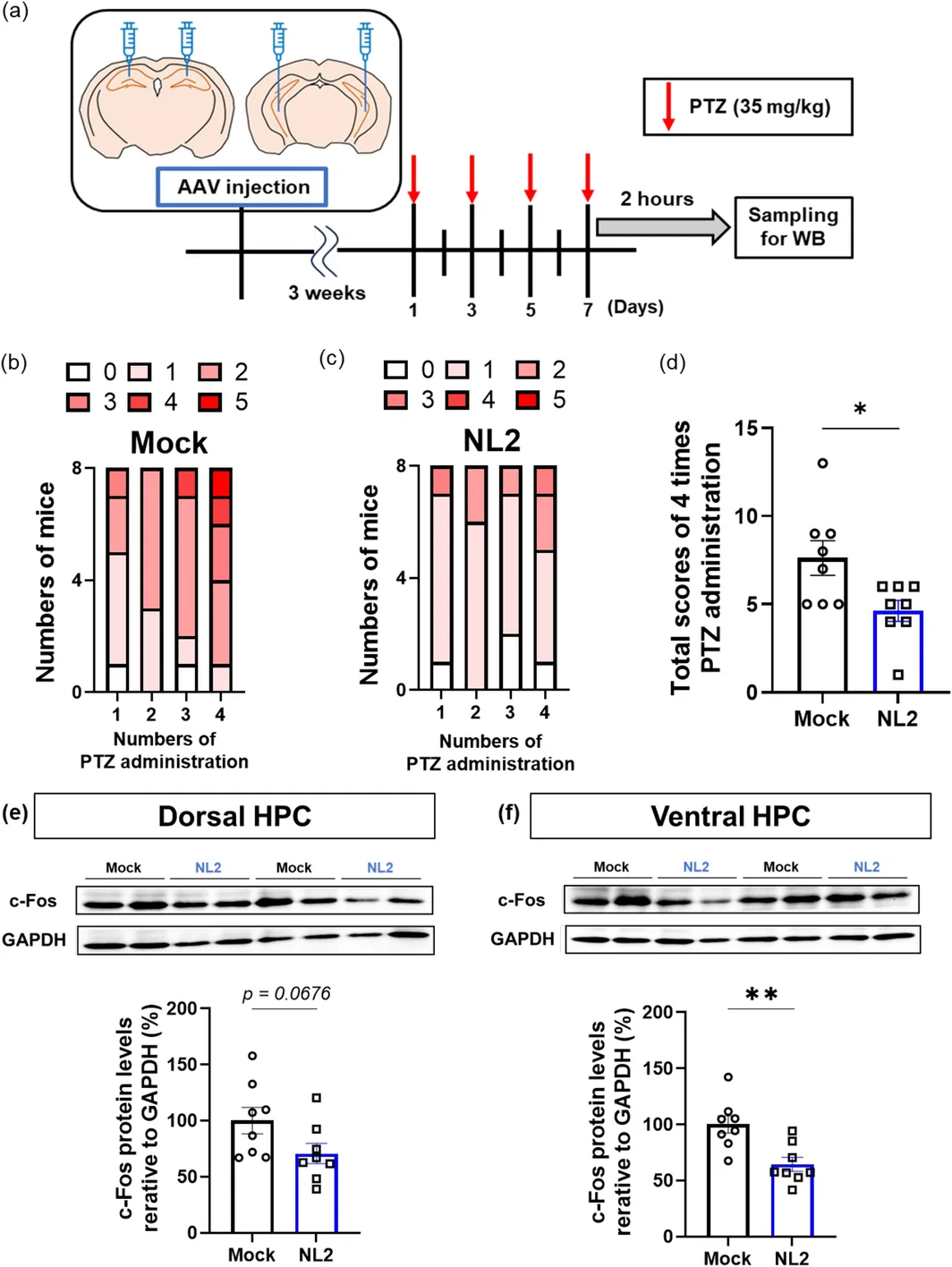
NL2 expression reduces c‐Fos protein in the HPC. (a) Experimental design. Three weeks after AAV vector injection, mice were intraperitoneally administered with PTZ (35 mg/kg) four times every other day, and HPC tissues were collected 2 h after the final PTZ administration. (b, c) Distribution of seizure scores over PTZ administrations in the (b) AAV‐mock and (c) AAV‐NL2 groups (*n* = 8 per group). (d) Cumulative scores over PTZ administrations (*n* = 8 per group; *p < 0.05, Mann–Whitney *U* test). (e, f) Representative western blots quantification of c‐Fos and GAPDH in the (e) dorsal (*n* = 8 per group; unpaired *t*‐test) and (f) ventral HPC (*n* = 8 per group; **p < 0.01, unpaired *t*‐test). Data are presented as the mean ± SEM. AAV, adeno‐associated virus; GAPDH, glyceraldehyde‐3‐phosphate dehydrogenase; HPC, hippocampus; NL2, neuroligin 2; PTZ, pentylenetetrazol.

## 4 Discussion

We demonstrated that AAV vector‐mediated NL2 expression in the HPC effectively attenuated PTZ‐induced seizure progression and significantly reduced seizure susceptibility, with NL2‐treated mice being more likely to exhibit lower seizure scores than the AAV‐mock group. This improvement was accompanied by a reduction in c‐Fos expression, only in the ventral HPC, following repeated PTZ administration. These results suggest that the intra‐HPC NL2 gene delivery can attenuate the development of induced seizures and alleviate neuronal hyperexcitation through local reinforcement of GABAergic inhibition. Although PTZ is administered systemically and acts broadly across the brain, the HPC is a particularly vulnerable region in PTZ‐induced seizure models [41, 42, 43].

PTZ inhibits GABA_A_ receptors at a domain overlapping with the picrotoxin site by increasing closed‐state duration and reducing open probability of chloride channels [44]. This reduction in GABAergic tone shifts the local excitation–inhibition balance toward hyperexcitability. In this study, intra‐HPC delivery of NL2 may have provided sufficient inhibitory capacity to counteract the PTZ‐induced increase in excitability. Previous studies have shown that NL2 maintains the dense clustering of GABA_A_ receptors at inhibitory synapses by stabilizing the postsynaptic gephyrin scaffold [21, 45, 46]. Notably, disruption of NL2 causes nanoscale dispersion of GABA_A_ receptors away from presynaptic release sites, reducing synaptic transmission efficiency without altering total receptor numbers [46]. Even when PTZ reduces channel open probability, an NL2‐dependent increase in the local synaptic density of GABA_A_ receptors may preserve the net inhibitory current. This could maintain effective GABAergic inhibition sufficient to compensate for the pharmacological blockade, thereby both preventing the initiation and delaying the progression of local seizures.

Interestingly, the suppression of neuronal activity by NL2 was more pronounced in the ventral HPC than in the dorsal HPC. The ventral HPC, which is homologous to the anterior HPC in humans [47, 48, 49], is recognized for its higher seizure susceptibility compared to the dorsal HPC [50, 51]. In a chronic temporal lobe epilepsy model with pilocarpine, the ventral hippocampal formation acts as a primary epileptogenic zone, whereas the dorsal HPC serves as a secondary site [52]. Moreover, following status epilepticus induced by soman, an organophosphate nerve agent, the ventral HPC and amygdala were more vulnerable to neuronal damage compared with the dorsal HPC [53]. Although dorsal–ventral differences have not been systematically characterized in the PTZ model, given this ventral predominance in seizure generation and susceptibility, the more pronounced NL2‐mediated reduction in c‐Fos expression in the ventral HPC may reflect a preferential stabilization of ictogenic limbic networks that are critically involved in PTZ‐induced seizures. However, the lack of a significant correlation between the c‐Fos expression and seizure scores indicates that the c‐Fos expression does not directly account for the severity of seizure activity. Thus, reduced neuronal activation indicated by c‐Fos expression should be considered supportive, but not definitive, evidence for the anticonvulsant effects of NL2 expression.

We assessed seizure susceptibility using ordinal logistic regression, which can model the relationships between several objective variables, represented as ordinal scales, and explanatory variables. This analysis has been used to estimate the therapeutic effects of a thrombolytic drug on the stroke severity scores [54]. Here, it applies to the association between ordinal variables, such as seizure scores and the protective effects of NL2 expression. Our analyses suggest that intra‐HPC injection of AAV‐NL2 can decelerate symptom progression caused by repeated PTZ administration.

We previously demonstrated that systemic administration of a tyrosine mutant AAV9/3 vector expressing NL2 under the control of synapsin 1 promoter suppresses seizures in a spontaneous epilepsy EL mouse model [25]. In the present study, we administered the AAV2 vector directly into brain parenchyma. Local administration to the brain enables targeted gene delivery confined to the vicinity of epileptic foci, minimizing side effects, as well as efficient gene therapy for spatiotemporally discrete, epileptic foci, such as hamartomas.

Only male mice were used in this study. Female rodent is reported to be more sensitive to PTZ than male [55, 56]. The AAV‐NL2 has a limited influence for seizure and female stronger sensitivity may mask the anti‐seizure effects. Our findings from male mice may not extend to female animals or humans so that it is essential to include of female mice as a future experimental direction.

## 5 Conclusion

We revealed that AAV vector‐mediated NL2 expression in the HPC attenuates seizure progression induced by repeated PTZ administration. NL2 expression also suppressed neuronal activation in the HPC. These findings suggest a potential role of NL2 in seizure suppression.

## Author Contributions

All the authors contributed to the conception and design of this study. Material preparation, data collection, and analysis were performed by Tomohiro Tokutake, Ayano Murayama, and Yuto Mitsuda. Data analysis was performed by Akane Hara, Jun Yokose, Yusuke Yano, and Yuki Shigetsura. AAV vector production was performed by Mika Ito, Naomi Takino, and Shin‐Ichi Muramatsu. Tomohiro Tokutake, Shinichi Kumagai, and Kuniko Shimazaki drafted the manuscript. The manuscript has been revised to its final version by Shin‐Ichi Muramatsu and Atsumi Nitta.

## Ethics Statement

The animal experimental protocols were approved by the Animal Care and Use Committee of the University of Toyama (Approval Number: A2025PHA14), and the experiments were performed in accordance with the Institutional Animal Experiment Handling Rules of the University of Toyama. The number of animals used was carefully estimated and maintained at the minimum necessary level for meaningful data interpretation. The experiments were approved by the Board of Safety Committee for Recombinant DNA Experiments at the University of Toyama (G2025PHA‐06).

## Conflicts of Interest

Shin‐Ichi Muramatsu has equity with the ONODERA GT Pharma Inc., which commercializes AAV vectors for gene therapy. All other authors declare no competing financial interests.

## Supporting information


**Figure S1:** (a) Representative image showing gephyrin (red), NL2 (green), DAPI, and merged expression at HPC treated with AAV‐NL2. Scale bars: 10 μm. (b) All membrane images of chemiluminescence and colorimetric in the HPC of AAV‐mock and AAV‐NL2 groups.
**Figure S2:** (a, b) All membrane images of chemiluminescence and colorimetric in the (a) dorsal and (b) ventral HPC.
**Figure S3:** (a) Kaplan–Meier survival curves in the mock and NL2 group (mock group: *n* = 15; NL2 group: *n* = 15; *p* = 0.0516; Log‐rank test). (b) Latency of seizure (minutes) in each number of administrations (mock group: *n* = 9; NL2 group: *n* = 12 at sixth PTZ administration; *p < 0.05; main effect of administration numbers: *F*_9, 201_ = 4.357, *p* < 0.0001; main effect of NL2: *F*_1, 201_ = 9.256, *p* = 0.0027; interaction effect numbers: *F*_9, 201_ = 0.7659, *p* = 0.6481, two‐way ANOVA followed by Bonferroni's post hoc test). (c) Body weight changes through PTZ administrations (main effect of administration numbers: *F*_1.179, 22.79_ = 17.66, *p* = 0.0002; main effect of NL2: *F*_1, 28_ = 1.371, *p* = 0.2514; interaction effect numbers: *F*_9, 174_ = 1.462, *p* = 0.1655, two‐way repeated measures ANOVA followed by Bonferroni's post hoc test). Data are presented as mean ± SEM.
**Figure S4:** (a, b) Correlation between seizure scores and relative c‐Fos levels in the (a) dorsal (*r* = 0.3254, *p* = 0.2176) (*n* = 8 per group; Spearman's *r*) and (b) ventral HPC (*r* = 0.1649, *p* = 0.5391) (*n* = 8 per group; Spearman's *r*).

## Data Availability

Data presented in this manuscript are available from the corresponding author upon request.
