# Supplementary material for: Adeno‐Associated Virus Vector‐Mediated Expression of Neuroligin 2 in the Mouse Hippocampus Attenuates the Progression of Pentylenetetrazol‐Induced Seizures
Source: J Gene Med. 2026 Oct 7;28(10):e70116. doi: 10.1002/jgm.70116 (PMC13646667; doi:10.1002/jgm.70116)
Supplement: Supplementary file 1 [file JGM-28-e70116-s001.docx]

**Figure S1.**

**a** Representative image showing Gephyrin (red), NL2 (green), DAPI, and merged expression at HPC treated with AAV-NL2. Scale bars: 10 μm. **b** All membrane images of chemiluminescence and colorimetric in the HPC of AAV-mock and AAV-NL2 groups.

**Figure S2.**

**a, b** All membrane images of chemiluminescence and colorimetric in the (**a**) dorsal and (**b**) ventral HPC.

**Figure S3.**

**a** Kaplan-Meier survival curves in the mock and NL2 group (mock group: n = 15; NL2 group: n = 15; *p* = 0.0516; Log-rank test). **b** Latency of seizure (minutes) in each number of administrations (mock group: n = 9; NL2 group: n = 12 at sixth PTZ administration; **p* < 0.05; main effect of administration numbers: *F*_9, 201_= 4.357, *p* < 0.0001; main effect of NL2: *F*_1, 201_ = 9.256, *p* = 0.0027; interaction effect numbers: *F*_9, 201_ = 0.7659, *p* = 0.6481, two-way ANOVA followed by Bonferroni’s post-hoc test). **c** Body weight changes through PTZ administrations. (main effect of administration numbers: *F*_1.179, 22.79_ = 17.66, *p* = 0.0002; main effect of NL2: *F*_1, 28_ = 1.371, *p* = 0.2514; interaction effect numbers: *F*_9, 174_ = 1.462, *p* = 0.1655, two-way repeated measures ANOVA followed by Bonferroni’s post-hoc test). Data are presented as mean ± SEM.

**Figure S4.**

**a, b** Correlation between seizure scores and relative c-Fos levels in the (**a**) dorsal (*r* = 0.3254, *p* = 0.2176) (n = 8 per group; Spearman’s *r*) and (**b**) ventral HPC (*r* = 0.1649, *p* = 0.5391) (n = 8 per group; Spearman’s *r*).
